# Parametric Amplification of Acoustically Actuated Micro Beams Using Fringing Electrostatic Fields

**DOI:** 10.3390/mi15020257

**Published:** 2024-02-09

**Authors:** Stella Lulinsky, Ben Torteman, Bojan R. Ilic, Slava Krylov

**Affiliations:** 1 School of Mechanical Engineering, Faculty of Engineereing, Tel Aviv University, Ramat Aviv, Tel Aviv 69978, Israel; stella64@tauex.tau.ac.il (S.L.); bentorteman@gmail.com (B.T.); 2 Center of Nanoscale Science and Technology, National Institute of Standards and Technology, Gaithersburg, MD 20899, USA; robert.ilic@nist.gov

**Keywords:** parametric amplification, MEMS, electrostatic actuation, acoustic sensor, cantilever

## Abstract

We report on theoretical and experimental investigation of parametric amplification of acoustically excited vibrations in micromachined single-crystal silicon cantilevers electrostatically actuated by fringing fields. The device dynamics are analyzed using the Mathieu–Duffing equation, obtained using the Galerkin order reduction technique. Our experimental results show that omnidirectional acoustic pressure used as a noncontact source for linear harmonic driving is a convenient and versatile tool for the mechanical dynamic characterization of unpackaged, nonintegrated microstructures. The fringing field’s electrostatic actuation allows for efficient parametric amplification of an acoustic signal. The suggested amplification approach may have applications in a wide variety of micromechanical devices, including resonant sensors, microphones and microphone arrays, and hearing aids. It can be used also for upward frequency tuning.

## 1. Introduction

Parametric amplification plays an important role in resonant micro and nanoelectromechanical (MEMS/NEMS) sensors due to its ability to bust vibrational amplitudes, improve frequency stability, and squeeze noise, therefore improving sensitivity [1,2,3,4,5,6,7,8,9,10]. Parametric amplification (also often referred to as parametric pumping [2,8,10,11]) of an external harmonic signal is achieved by time modulating the system’s intrinsic, usually stiffness, parameters. On the most basic level, the dynamics of the devices liable to parametric amplification are described by the (linear or nonlinear) nonhomogeneous counterpart of the Mathieu equation containing an additional forcing term [1,2,3,12,13,14]. As opposed to the case of parametric resonance, in the parametric amplification regime, the amplitude of the time-dependent stiffness modulation is lower than the parametric resonance threshold in a damped system. In microdevices, modulation of the (effective) spring constant is relatively straightforward due to the intrinsic nonlinearity of the actuation forces. The parametric pumping was realized using mainly electrostatic [1,4,5,6,10,15,16] but also piezoelectric [1,9] or magnetic [7] actuation. Interestingly, the realization of a linear direct forcing in MEMS/NEMS structures could be more challenging than the parametric amplification itself. For instance, the use of electrostatic actuation for the direct harmonic driving [1,4,5,6] introduces nonlinearity and may alter the spectral characteristics of the structure even when the vibrational amplitudes are very small (compared to the distance between the electrodes). While integration of piezoelectric materials allows achieving linear actuation and high amplitudes [2], it requires the deposition of additional layers, which broadens the vibrational characteristics of the device, complicates the fabrication process, and may induce undesired residual stresses. As an alternative, inertial excitation through mounting the device on an external piezoelectric shaker is widely used for linear mechanical dynamic characterization [3]. To realize parametric amplification in microdevices, the electrical signal should be provided in addition to the inertial actuation. Since a device mounted on a vibrating surface cannot be electrically connected by micromanipulators (probes) or probe cards, a combined inertial and eclectic actuation scenario requires a certain level of device integration, such as wire bonding and packaging. For the same reason, it is not suitable for wafer-level testing.

In this work, we use forces of different natures for parametric amplification and the linear external harmonic signal. Specifically, electrostatic actuation associated with fringing electrostatic fields is used for stiffness modulation, while acoustic pressure is the source of linear noncontact mechanical excitation. Acoustic actuation is distinguished by a linear dependence between the voltage applied to the speaker and the amplitudes of the excited vibrations of the microstructure. Generally, steadily growing interest in the interaction between microstructure and acoustic fields was motivated mainly by the development of MEMS microphones [17,18,19,20,21,22], speakers [23], hearing aids [24,25], and bio-inspired highly directional acoustic sensor arrays [26,27,28,29]. Understanding the influence of environmental noise on structural response is important also for the development of microaccelerometers and gyros [30,31]. However, the acoustic field is rarely used for actuation. Few works reported on resonant acoustic excitation of optical microscanners [32] or micro cantilevers [33,34]. Here, we use acoustic fields as a means for the linear mechanical excitation of a parametrically amplified device. The goal of this work is twofold. First, we show that acoustic excitation through an external speaker can serve as a convenient and efficient experimental technique for mechanical dynamic characterization of unpackaged and even not electrically connected resonant MEMS. We emphasize that the acoustic excitation is explored here not as a means to replace commonly implemented characterization approaches of acoustic devices such as microphones but as a convenient, noninvasive, contactless tool providing a linear harmonic excitation signal to an unpackaged MEMS device operated at resonance. Second, we demonstrate that the use of the fringing electrostatic fields allows efficient parametric amplification of an acoustic signal.

## 2. Methodology

### 2.1. Device Architecture

**Figure 1:**
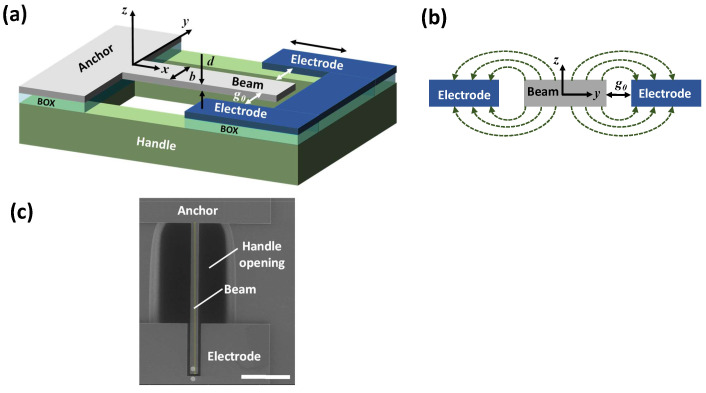
(**a**) Schematics of the device. (**b**) Schematics of the symmetric fringing fields. The section of the beam and of the electrodes by a plane parallel to the yz plane is shown. (**c**) Scanning electron micrograph (SEM) of the fabricated device. The scale bar is 100 µm.

Our device [35,36] consists of a cantilever of a length *L*, width *b*, and thickness *d*, as seen in Figure 1a. A planar electrode of length $L_{e}$ surrounding the beam is located symmetrically at two sides of the beam, at the distance $g_{0}$. The beam can freely deflect in the out-of-plane (z) direction, whereas its in-plane (y) deflection is negligibly smaller due to the high width-to-thickness ratio (b≫d). To allow large unobscured vibrations and prevent stiction, an opening is created in the substrate under the beam. Since both the cantilever and the electrode are fabricated from the same wafer layer, the electrostatic field is fully symmetric and the resultant electrostatic force is zero in the initial undeformed configuration, as seen in Figure 1b. In the deformed state, the distributed electrostatic force, arising from asymmetries of the fringing fields, acts in a direction opposite to the beam’s deflection and effectively serves as a restoring force. Application of a time-dependent pumping (parametric) voltage $V_{p}\left(\hat{t}\right)=V_{dc}+V_{ac}\cos\left({\hat{\omega }}_{p}\hat{t}\right)$ to the electrode results in parametric excitation of the beam, as reported in [35,36]. Here, our investigation is extended to the case of simultaneous mechanical acoustic and parametric electrostatic excitation.

Using deep reactive ion etching (DRIE), cantilevers and electrodes were fabricated from a 3 µm thick silicon device layer using a silicon on insulator (SOI) wafer with a 4 µm thick buried thermal silicon dioxide (BOX) layer. DRIE was used also to etch a cavity within the handle of the wafer. The devices were released using a vapor hydrofluoric acid (HF) process. The fabricated device is shown in Figure 1c. The nominal (as designed) and measured dimensions of the structure are listed in Table 1.

### 2.2. Model

The cantilever is modeled as a Euler–Bernouli beam. The distributed electrostatic force associated with the asymmetry of the fringing fields between the electrode and the beam was evaluated according to the procedure presented in [35]. The Galerkin decomposition (with the fundamental mode of the cantilever’s free undamped vibrations serving as the base functions) was used to obtain a single degree of freedom (SDOF) model, which, despite its simplicity, captures the beam dominant dynamics. In the framework of the SDOF approximation, the motion of the beam is governed by the nondimensional equation

(1) $$\begin{array}{c} \ddot{q}+\frac{\omega_{0}}{Q}\dot{q}+\omega_{0}^{2}q+\beta V_{p}^{2}\frac{\sinh(\sigma q)}{\cosh^{p}\left(\sigma q\right)}=\eta V_{spkr}\cos\left(\omega t+\phi \right) \end{array}$$

Here, q=q(t) is the nondimensional time-dependent end point deflection of the beam, $\omega_{0}=1.875^{2}$ is the nondimensional fundamental mode frequency of the unforced ideally clamped cantilever, *Q* is the quality factor, β is a voltage parameter, $V_{p}=V_{p}\left(t\right)$ is the parametric (pumping) voltage, σ and *p* are the electrostatic force fitting parameters obtained in [37] based on the numerical solutions of the electrostatic problem for the same nominal beam cross-section geometry b×d, and the distance to the electrode of $g_{0}=3\;$µm. In addition, $V_{spkr}$ is the amplitude of the voltage signal supplied to the speaker and ω and ϕ are the nondimensional frequency and phase of the driving speaker signal, respectively. The modal coefficient η is associated with the actuating acoustic pressure and can be found using multi-physics finite element simulations or experimentally. Hereafter, overdot denotes the derivative with respect to nondimensional time *t*. Nondimensional quantities are defined in Table 2, and the adopted numerical values of the material properties, the modal coefficients, and the electrostatic force fitting parameters are listed in Table 3.

In accordance with Equation (1), due to the assumption of the field symmetry with respect to the z=0 plane in the undeformed configuration, Figure 1b, the fringing-field’s electrostatic force is zero at q=0, affects only the effective mechanical stiffness, and does not contribute to the direct driving of the device. However, in practice, additional “parasitic” direct electrostatic forcing may emerge and may have a certain effect at the resonant operation [35,36]. These “parasitic” forces can be attributed to the asymmetry of the electric fields due to the imperfections in the beam geometry (for example, the beam’s initial curvature due to the residual stress) as well as to the contribution of the fringing fields emerging from the substrate or the side walls of the cavity etched into the substrate. The third-order Taylor-series expansion (around q=0) of the electrostatic force in Equation (1) completed by the “parasitic” direct electrostatic forcing terms yields

(2) $$\begin{array}{c} \ddot{q}+\frac{\omega_{0}}{Q}\dot{q}+\left[\omega_{e}^{2}+\gamma_{1}\cos\left(\omega_{p}t\right)+\gamma_{2}\cos\left(2\omega_{p}t\right)\right]q\\ \;-\left[\alpha_{0}+\alpha_{1}\cos\left(\omega_{p}t\right)+\alpha_{2}\cos\left(2\omega_{p}t\right)\right]q^{3}\\ \;=\eta V_{spkr}\cos\left(\omega t+\phi \right)+\mu_{0}+\mu_{1}\cos\left(\omega_{p}t\right)+\mu_{2}\cos\left(2\omega_{p}t\right) \end{array}$$

where

(3) $$\begin{array}{c} \gamma_{1}=2\beta \sigma V_{dc}V_{ac}\;,\;\gamma_{2}=\frac{\beta \sigma V_{ac}^{2}}{2}\\ \alpha_{0}=V_{\mathrm{eff}}^{2}\beta \left(\frac{p}{2}-\frac{1}{6}\right)\sigma^{3}\;,\;\alpha_{1}=2V_{dc}V_{ac}\beta \left(\frac{p}{2}-\frac{1}{6}\right)\sigma^{3}\;,\\ \alpha_{2}=V_{ac}^{2}\frac{\beta }{2}\left(\frac{p}{2}-\frac{1}{6}\right)\sigma^{3}\;,\;\omega_{e}=\sqrt{\omega_{0}^{2}+\beta \sigma V_{\mathrm{eff}}^{2}} \end{array}$$

Here, $\omega_{e}$ is the electrostatically detuned (effective) fundamental mode frequency, $\gamma_{i}$ and $\alpha_{i}$ are the stiffness modulation amplitudes of the linear and cubic parametric terms, respectively, and the index i=1,2 indicates one of the time scales (i.e., $\omega_{p}$ and $2\omega_{p}$). In addition, $\alpha_{0}$ is the coefficient of the cubic nonlinearity and $V_{\mathrm{eff}}^{2}=V_{dc}^{2}+V_{ac}^{2}/2$ is the effective electrostatic steady (effective dc) voltage. The direct “parasitic” electrostatic actuation is accounted for, in the most general form, by the three last terms on the right-hand side of Equation (2), where the (highly uncertain and generally depending on the voltages $V_{dc}$ and $V_{ac}$) amplitudes $\mu_{0}$, $\mu_{1}$, and $\mu_{2}$ are associated with the static, single frequency $\omega_{p}$, and double-frequency $2\omega_{p}$ driving, respectively.

The presence of the two stiffness modulation time scales in Equation (2) suggests that there are at least two scenarios at which primary parametric excitation or parametric amplification can be achieved. Specifically, either the pumping voltage frequency is around the cantilever resonant frequency, i.e., $\omega_{p}\approx \omega_{e}$, or the operation is at twice the resonant frequency, in the vicinity of $\omega_{p}\approx 2\omega_{e}$. In both cases, for the parametric amplification, the driving frequency of the acoustic force is set to be $\omega \approx \omega_{e}$ [1]. In the first scenario, the amplitude of the stiffness modulation at the double frequency is parameterized by $\gamma_{2}$ in Equation (2) (the $\gamma_{1}$ term may have contribution at higher modulation amplitudes through the secondary parametric excitation mechanism at $\omega_{p}\approx \omega_{e}$ [38]). However, since, in this case, the direct resonant driving is due to a combination of the acoustic excitation and the “parasitic” electrostatic drive with (generally uncertain) amplitude $\mu_{1}$, this scenario is less convenient for the investigation of the parametric amplification. Therefore, to reduce the effect of the “parasitic” electrostatic forces to a minimum and to obtain a “pure” parametric amplification of the acoustic signal, in our analysis, we implement the second scenario and avoid any electrostatic actuation within the primary resonance bandwidth. Specifically, we only analyze a scenario in which the pumping (parametric) frequency is set to twice the acoustic drive (speaker) frequency, i.e., $\omega_{p}=2\omega$. In this case, all the direct electrostatic driving terms in Equation (2) are nonresonant, and their contribution is minor. In addition, as this work is primarily focused on the parametric amplification, the effective stiffness modulation amplitude was kept below the parametric resonance threshold [38] (see [7,12] for nonlinear parametric amplification within the boundaries of the parametric resonance regime).

To obtain an approximate solution for Equation (2), (where we neglect all the nonresonant direct electrostatic driving terms with the amplitudes $\mu_{0},\mu_{1},\mu_{2}$) we employ the method of averaging [38]. To this end, we normalize the nondimensional time and nondimensional frequency with respect to the detuned effective fundamental mode frequency

(4) $$\begin{array}{cc} \; & \tau =t\omega_{e},\;\Omega =\omega /\omega_{e},\;\Omega_{p}=\omega_{p}/\omega_{e}=2\Omega \end{array}$$

By limiting our consideration to the small vibrational amplitudes, we introduce a small bookkeeping parameter ϵ, which yields

(5) $$\begin{array}{c} \frac{d^{2}q}{d\tau^{2}}+2\epsilon \zeta \frac{dq}{d\tau }+\left[1+\epsilon \gamma_{1}^{★}\cos\left(\Omega_{p}\tau \right)+\epsilon \gamma_{2}^{★}\cos\left(2\Omega_{p}\tau \right)\right]q\\ -\left[\epsilon \alpha_{0}^{★}+\epsilon \alpha_{1}^{★}\cos\left(\Omega_{p}\tau \right)+\epsilon \alpha_{2}^{★}\cos\left(2\Omega_{p}\tau \right)\right]q^{3}\\ =\epsilon \eta^{★}V_{spkr}\cos\left(\Omega \tau +\phi \right) \end{array}$$

where ${\left(\;\right)}^{★}=\left(\;\right)/\omega_{e}^{2}$ and $2\zeta =\omega_{0}/\left(\omega_{e}Q\right)$. A similar system has been studied in [13] to describe the dynamics of a base-excited beam.

In the framework of the averaging method, we introduce a coordinate transformation [38]

(6) $$\begin{array}{c} q(\tau )=X(\tau )\cos(\Omega \tau )+Y(\tau )\sin(\Omega \tau )\;,\\ \frac{dq(\tau )}{d\tau }=-X\left(\tau \right)\Omega \sin\left(\Omega \tau \right)+Y\left(\tau \right)\Omega \cos\left(\Omega \tau \right) \end{array}$$

where X(τ) and Y(τ) satisfy the implicit constraint

(7) $$\begin{array}{c} \frac{dX}{d\tau }\cos\left(\Omega \tau \right)+\frac{dY}{d\tau }\sin\left(\Omega \tau \right)=0 \end{array}$$

and introduce a frequency-detuning parameter

(8) $$\begin{array}{c} \Delta =\frac{\Omega -1}{\epsilon } \end{array}$$

By subsisting Equations (6) and (8) into Equation (5), solving for the dynamic variables (using Equation (7)), and averaging over one time period (2π/Ω), we obtain the slow flow equations

(9) $$\begin{array}{c} \frac{dX}{d\tau }=\frac{1}{16}\epsilon [\left(4\alpha_{1}^{★}-6\alpha_{0}^{★}-\alpha_{2}^{★}\right)Y^{3}+\left(3\alpha_{2}^{★}-6\alpha_{0}^{★}\right)X^{2}Y\\ \;-16\zeta X-\left(4\gamma_{1}^{★}+16\Delta \right)Y+8\eta^{★}\sin\left(\phi \right)]+O\left(\epsilon^{2}\right)\;,\\ \frac{dY}{d\tau }=\frac{1}{16}\epsilon [\left(6\alpha_{0}^{★}+4\alpha_{1}^{★}+\alpha_{2}^{★}\right)X^{3}+\left(6\alpha_{0}^{★}-3\alpha_{2}^{★}\right)XY^{2}\\ \;+\left(16\Delta -4\gamma_{1}^{★}\right)X-16\zeta Y+8\eta^{★}\cos\left(\phi \right)]+O\left(\epsilon^{2}\right) \end{array}$$

The steady-state solution of Equation (9) corresponding to the steady-periodic responses of Equation (5) are attained by setting in Equation (9) all the time derivatives to zero and by solving numerically the resulting system of nonlinear algebraic equations for *X* and *Y*. It is convenient to convert the coordinates from Cartesian into polar (amplitude and phase) and to represent the response of the beam in the form

(10) q(τ)=acos(Ωt−ψ)

where $a=\sqrt{X^{2}+Y^{2}}$ is the beam’s steady-state amplitude and $\psi =\tan\left(Y/X\right)$ is phase. The gain metric of the parametric amplifier is defined as

(11) $$\begin{array}{c} G\left(\Delta =0\right)=\frac{a}{a_{REF}} \end{array}$$

where $a_{REF}={\left.a\right|}_{V_{p}\;=\;0}$ is the reference steady-state amplitude of the beam in the absence of the electrostatic actuation, when $V_{p}=0$. Since, in the adopted actuation scenario, the parametric terms in Equation (5) are proportional either to $V_{ac}$ (coefficient $\gamma_{1}^{★}$) or to $V_{ac}^{2}$ (coefficient $\gamma_{2}^{★}$), the zero parametric amplification scenario can be realized even for $V_{dc}\neq 0$, if $V_{ac}$ is set to zero. In this case ($V_{dc}\neq 0,\;V_{ac}=0$), the ac acoustic and the dc electrostatic actuation are uncoupled. Namely, the acoustic field is the source of the linear harmonic drive at the frequency Ω while the dc voltage is used only for the (up)tuning of the device’s effective natural frequency. However, for simplicity, in the present work, we adopt the amplitude of the beam driven solely by the acoustic fields (at $V_{p}=0$) to serve as the reference amplitude.

**Figure 2:**
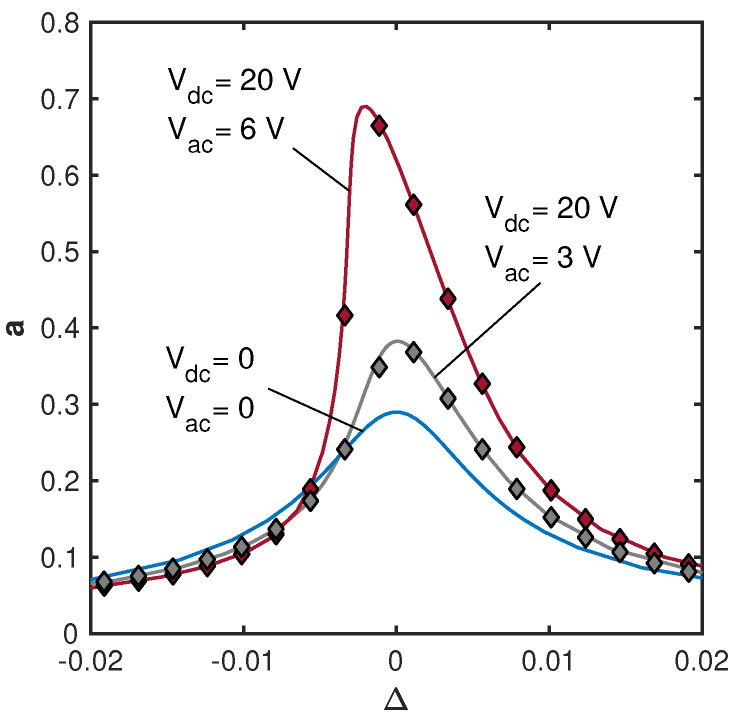
Model results—frequency responses of the cantilever with the nominal dimensions, Table 1, obtained for $\omega_{p}=2\omega$, for three different combinations of the steady (dc) and time-harmonic (ac) amplitudes $V_{dc},\;V_{ac}$ of the parametric voltage $V_{P}$, the speaker’s voltage $V_{spkr}=10$ V, ϕ=0, and Q=100. Other parameters used in calculations are detailed in Table 3. Solid lines correspond to the approximate asymptotic model, Equation (9), and markers correspond to the numerical integration of Equation (2), where $\mu_{0}=\mu_{1}=\mu_{2}=0$.

**Figure 3:**
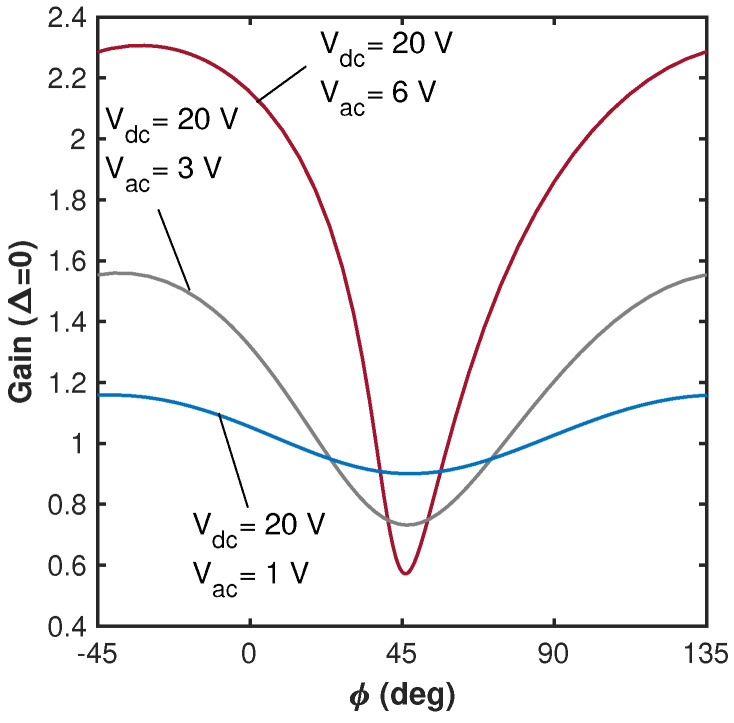
Model results—the phase dependence of the amplifier gain (at Δ=0) (where ϕ is the phase between the acoustic driving and the pumping (parametric) voltage signals, Equation (2)) at various pumping voltages obtained for $\omega_{p}=2\omega$. The geometric and operational parameters are identical to those used in Figure 2.

Figure 2 presents the frequency responses for various parametric voltage values below the parametric resonance threshold. The calculations were carried out for the nominal dimensions of the beam, Table 1, and for the material and actuating forces parameters listed in Table 3. The modal acoustic force coefficient η was chosen such that for the adopted actuation parameters $V_{spkr}=10$ V, ϕ=0, Q=100 (and consistently with the experiments), the resulting resonant amplitude deflection would be 0.9 µm. Comparison between the vibrational amplitudes obtained using the approximate asymptotic model, Equation (9), with the results of the direct numerical integration of Equation (2) show good agreement between the two. In our numerical solution, the equation of motion Equation (2) completed by zero initial conditions was solved numerically for several values of the driving ω and pumping $\omega_{p}$ frequencies. The computation time was chosen to be long enough to reach the steady state; the vibrational amplitude was obtained by averaging the amplitudes of the last 25% of the time history. Figure 2 illustrates that for the same magnitude of the acoustic driving signal (associated with the acoustic force coefficient η), the response amplitude grows with the increase in the electrostatic pumping voltage. At larger amplitudes, due to the nonlinearity of the electrostatic forces, Equation (1), a certain deviation of the resonant curve from the linear Lorentzian shape is observed. The phase dependence of the amplifier gain is illustrated in Figure 3 for the same system parameters as in Figure 2. Since the pumping electrostatic voltage affects not only the amplitudes of stiffness modulation but also the coefficients of the cubic nonlinearity (see Equation (3)), the value of the phase corresponding to the maximal gain varies with the pumping voltage [12].

In addition to the SDOF model analysis, we also used Comsol Multi-physics software (version 6.2) for assessing the displacement amplitudes of the beam interacting with the acoustic pressure. The analysis was carried out in two steps. First, the purely acoustic problem was solved (the Pressure Acoustic Analysis module was used for this purpose) to obtain the pressure distribution at a distance of ≈5 cm from the piston-type sound source. This distance is approximately equal to the value used in the experiment. At the second step, we assumed an incident plane acoustic wave with the pressure of 12 Pa and calculated the cantilever displacements using an Acoustic Structure Interaction module in Comsol. The results indicate that in this actuation scenario, the detectable deflections with maximal end point amplitudes within sub-micrometer range can be achieved. The goal of this analysis was to obtain only a coarse estimation of the cantilever amplitudes, which can be expected under acoustic actuation. The acoustic model did not incorporate all the details of the experiment. For example, in the experiment, which was not carried out in an anechoic chamber, the incident acoustic wave was not fully planar, and its propagation direction was not perpendicular to the cantilever top surface. However, despite the essentially approximate character of the acoustic model, the order of magnitude of the measured beam’s amplitudes was generally consistent with the model predictions.

## 3. Experiment

### 3.1. Experimental Setup

**Figure 4:**
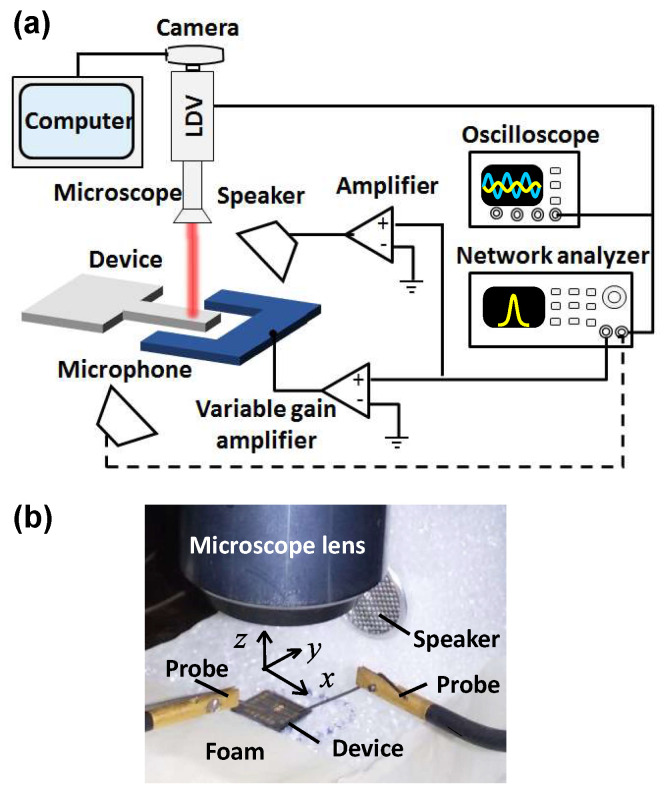
(**a**) Schematics of the experimental setup. The dashed line shows the connection of a microphone used for the monitoring of the acoustic field. (**b**) The device is mounted onto a vibroisolating foam and electrically connected by microprobes. The die size is ≈1 cm × 1 cm. The beam is oriented along the *x* axis (see Figure 1a).

The schematics of the experimental setup are illustrated in Figure 4a. The device was mounted on a chuck of a wafer prober. The out-of-plane response of the beam was registered using a single-beam laser Doppler vibrometer (LDV). The entire experimental setup including the LDV adapter was mounted on an anti-vibration table. To eliminate the influence of undesired vibrations of the die, the chip and the speaker were placed onto a vibroisolating foam, Figure 4b. Note that the same acoustic transducer (400ST/R160 by Midas Components Ltd., Great Yarmouth, UK) was used as the speaker and as the microphone. The positions and the orientations of the cantilever and the speaker were dictated by the limitations of the experimental setup. The speaker was not located above the beam (not to obscure the LDV laser beam coming from the microscope’s lens), and the incoming pressure wave was not normal to the cantilever upper surface. Instead, the speaker was located in such a way that the line connecting the speaker’s center and the cantilever (oriented along the *x*-axis, Figure 4b) made an angle of ≈$30^{0}$ with respect to the die (xy) plane. The beam was grounded and the electrode was connected to a voltage source using micromanipulators (probes). Experiments of several types were carried out. In the first series of the experiments, the setup illustrated in Figure 4a was used. The sinusoidal, zero offset (dc) voltage signal provided by a network analyzer was split into two channels. The first channel, connected to a fixed × 20 gain amplifier, supplied a signal to the speaker located at the distance of ≈5 cm from the device. The second channel, connected to a variable gain (up to × 150) amplifier, supplied a signal to the electrode. In this arrangement, the frequencies of the voltage signals supplied to the speaker and to the electrode were the same and around the cantilever resonant frequency, such that $\omega =\omega_{p}\approx \omega_{e}$. The output of the LDV was fed back into the network analyzer. The velocity time history of the LDV output was monitored by an oscilloscope. We also used a modified setup, which incorporated a two-channel wave generator to be able to provide independently two separate voltage signals to the speaker and the electrode. In this case, the output of the LDV was fed back to a real-time spectrum analyzer. This setup was used to investigate the dependence between the beam amplitude on the phase between the two (acoustic and electrostatic) signals.

### 3.2. Experimental Results

**Figure 5:**
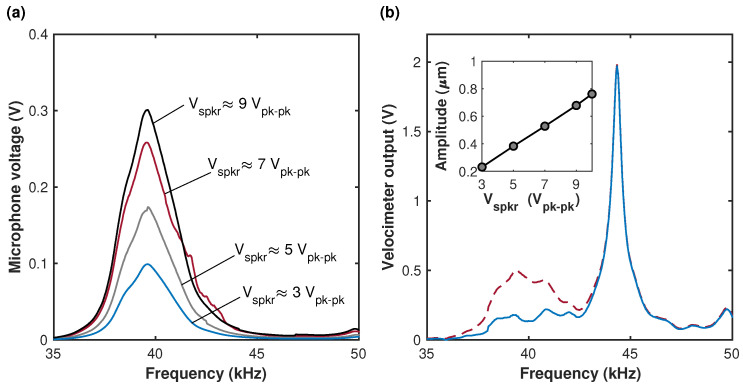
(**a**) Experimental frequency response of the acoustic transducer (of the microphone). Numbers correspond to the peak-to-peak voltage applied to the speaker. (**b**) Frequency response of the cantilever before (dashed line) and after (solid line) extraction of the normalized microphone output. The speaker voltage was $V_{spkr}\approx$ 9 $V_{pk-pk}$. The inset illustrates the dependence of the measured beam amplitude on the voltage applied to the speaker. The displacement error bars on the inset are 15 nm. The horizontal axis in (**a**,**b**) represents the frequency of the voltage signal supplied to the speaker.

First, the acoustic field produced by the speaker undergoing sinusoidal excitation within the interval of frequencies between ≈35 kHz and ≈50 kHz was characterized using another, identical to the speaker, acoustic transducer serving as a microphone and placed at a distance of a few cm from the speaker. The monitoring of the microphone output using a network analyzer (dashed line in Figure 4a) revealed a resonant peak at ≈39.61 ± 0.05 kHz, Figure 5a. Next, vibrations of the beam were induced acoustically by supplying the ac voltage to the speaker, and the resonant responses of the structure were investigated. In this experiment, the beam’s actuating electrode was disconnected from the voltage source, and the conditions of a pure acoustic excitation were realized. Two peaks are observed at the cantilever’s frequency curve—the first is associated with the speaker resonance, while the second, observed at ≈44.34 ± 0.05 kHz, corresponds to the cantilever resonance, Figure 5b. (The calculated resonant frequency of the ideally clamped cantilever with the nominal dimensions is $f_{0}=45.853$ Hz.) To distinguish between the spectral outputs of the speaker and of the beam, the re-scaled response of the speaker was subtracted from the LDV output, Figure 5b. We found that due to the difference of ≈4.74 kHz between the resonant frequencies of the speaker and of the cantilever, the dynamic characteristics of the speaker have only a minor influence on the spectral response of the microstructure. Acoustically excited amplitudes of the cantilever were estimated using the measured velocity time history by assuming a harmonic response and by subdividing the velocity amplitude by 2$\pi \hat{f}$, where $\hat{f}$ is the response frequency. The cantilever amplitude values in the range between ≈0.2 ± 0.015 µm and ≈0.9 ± 0.015 µm were obtained in the experiment and demonstrated linear dependence between the MEMS structure response and the voltage signal amplitude applied to the speaker, as shown in the inset of Figure 5b. These experimental results are consistent with the amplitudes provided by the numerical Comsol results.

**Figure 6:**
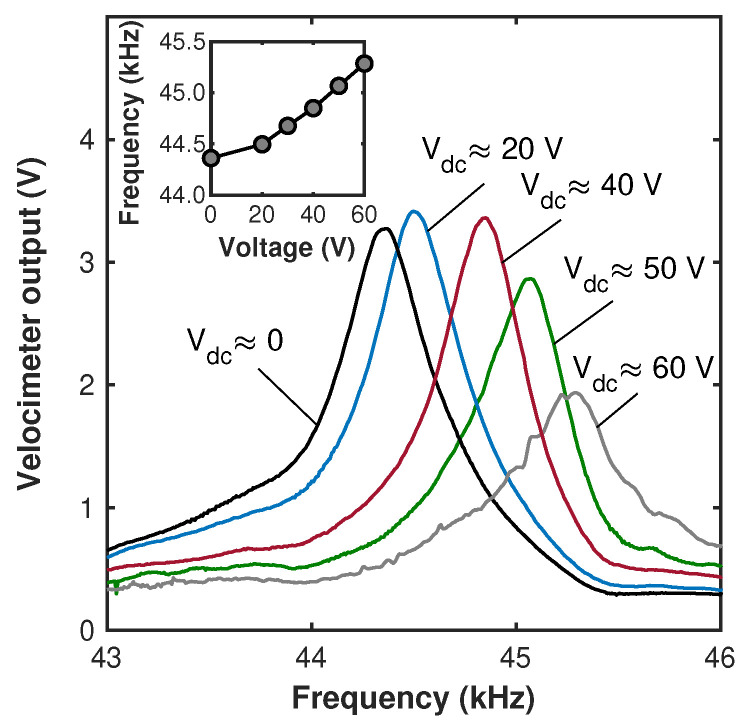
Experimental frequency response curves of the cantilever. Numbers correspond to the value of the dc voltage applied to the electrode. The voltage applied to the speaker was $V_{spkr}\approx$ 9 $V_{pk-pk}$. The inset depicts the frequency tuning curve—the dependence of the resonant frequency on the dc voltage. The error bars in the inset are 50 Hz.

In the framework of the frequency tuning experiment, the devices were operated simultaneously by the time-harmonic acoustic and time-independent steady (dc) electrostatic fringing fields (such that $V_{p}=V_{dc},\;V_{ac}=0$). Since the distributed electrostatic force acts in the direction opposite to the deflection of the beam, it serves as a positive effective spring. As a result, the stiffness increases with increasing voltage $V_{dc}$, as suggested by Equations (2) and (3). In our frequency tuning experiments, a harmonic voltage of $V_{spkr}$$\approx 5\;V_{pk-pk}$ was applied to the speaker and was the only source inducing the beam’s oscillations. The signal frequency was swept between ≈43 kHz and ≈47 kHz for a duration of ≈25 s. Simultaneously, a steady-state dc voltage $V_{dc}$ was applied to the electrode. The spectral responses of the cantilever and the resonant value of the velocity amplitudes corresponding to different values of the dc voltage were measured, Figure 6. By applying a steady dc voltage of up to ≈60 V, we demonstrate that the fundamental beam frequency, harmonically excited by an acoustic field, can be tuned up to ≈2.5 percent. This result is consistent with the frequency tuning values reported previously for similar beams [35,39]. Note that direct comparison of the measured relative frequency shift with the theoretical value $\Delta \omega \approx \beta \sigma V_{{\mathrm{eff}}^{2}}/\left(2\omega_{0}^{2}\right)$ predicted by the lumped model, Equations (2) and (3) (while neglecting the small possible contribution of the nonlinear terms to the tuning), is not fully applicable since the fitting parameters listed in Table 3 were derived in [37] for the electrostatic gap of $g_{0}=3\;$µm, which is smaller than the measured gap of $g_{0}\approx 5.6\;$µm, Table 1. As a result, the measured frequency shift is smaller than predicted by the model. By re-scaling the fitting parameter *a*, Table 3, under an assumption that the electrostatic force is roughly inversely proportional to the square of the distance between the electrodes, the frequency tuning of ≈3.3% at $V_{\mathrm{eff}}=60$ V was obtained.

**Figure 7:**
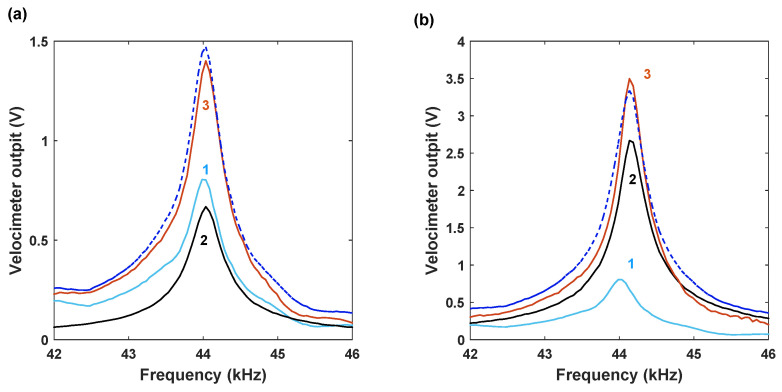
Experimental frequency curves illustrating excitation of the cantilever by the acoustic and the electrostatic fields. Curve 1 corresponds to the pure acoustic excitation $V_{dc}=V_{ac}$ = 0; curve 2 represents pure electrostatic excitation $V_{spkr}=0$; curve 3 is associated with the combined acoustic and electrostatic excitation. The dashed line represents the sum of the amplitudes corresponding to curves 1 and 2. (**a**) $V_{spkr}\approx 600$ m$V_{pk-pk},V_{ac}\approx 30$$V_{pk-pk}$; (**b**) $V_{spkr}\approx 600$ m$V_{pk-pk},V_{ac}\approx 60$$V_{pk-pk}$. In all the cases, $V_{dc}=0$ in the electrostatic signal.

Next, harmonic voltage signals $V_{spkr}\cos\left(\omega t\right)$ and $V_{p}=V_{ac}\cos\left(\omega t\right)$ were applied simultaneously to the speaker and the electrode, respectively. The steady component of the voltage supplied to the electrode was zero $V_{dc}=0$. The same frequency $\omega =\omega_{p}$ of the voltage signals supplied to the speaker and the electrode was swept in the vicinity of the cantilever effective resonance frequency $\omega_{e}$. Since, in this case, the beam undergoes simultaneously a direct harmonic acoustic forcing at the frequency ω and also parametric excitation by the time-varying electrostatic force at the frequencies of $\omega_{p}=\omega$ (the term proportional to $\gamma_{1}$ in Equation (2)) and of $2\omega_{p}=2\omega$ (the term proportional to $\gamma_{2}$), the case of a degenerate parametric amplification [1,2,3] is realized. However, the results shown in Figure 7a,b do not provide sufficient quantitative information regarding the role of each of the two (direct and parametric) excitation mechanisms in the combined effect and indicate that the demonstration of the parametric amplification in the framework of this operational scenario ($\omega_{p}=\omega \approx \omega_{e}$) can be challenging. The reason is the presence of the “parasitic” direct resonant driving term ($\mu_{1}\cos\left(\omega_{p}t\right)$ in Equation (2)) associated with the electrostatic signal. Our measurements show that, in the case of purely electrostatic driving (when $V_{spkr}=0$), the resonant response of the beam (the black lines in Figure 7) can be comparable or higher than that of the acoustically driven beam (the bright blue lines). The response due to the combined acoustic and electrostatic excitation (the orange lines) does not differ significantly from the summation of the “purely acoustic” and “purely electrostatic” responses (the dashed blue lines). In accordance with Equation (2), the parametric amplification of the direct “parasitic” electrostatic signal of generally unknown amplitude may also alter the response in the case of purely electrostatic excitation. Note also that although the acoustic and the electrostatic signals were supplied by the same voltage source and are of the same frequency, the phase between the signals may exist since they were passed through different voltage amplifiers. As a result, since the amplification gain is phase-dependent, Figure 3, the acoustic and the “parasitic” electrostatic signals may undergo different levels of amplification or even deamplification.

**Figure 8:**
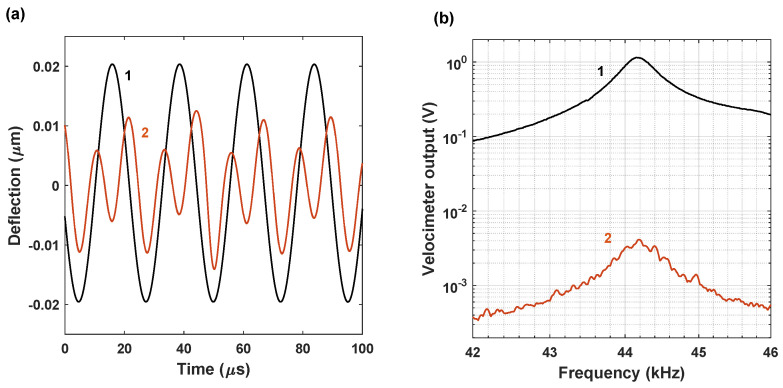
(**a**) Experimental time histories of the cantilever driven by the purely electrostatic ($V_{spkr}=0$) signal at the resonant frequency $\omega_{p}\approx \omega_{e}$ and twice this frequency $\omega_{p}\approx 2\omega_{e}$: curve 1 corresponds to $V_{dc}=0,\;V_{ac}=40$ $V_{pk-pk}$, and $\omega_{p}=$ 44.17 kHz; curve 2 corresponds to the multiplied by factor 50 response for the case of $V_{dc}=20$ V, $V_{ac}=40$ $V_{pk-pk}$, and $\omega_{p}=$ 88.34 kHz. (**b**) Frequency curves corresponding to the same voltages. The frequency axis corresponds to the frequencies of the beam’s vibratory response.

Therefore, to reduce the influence of the parasitic direct electrostatic driving, all the further experiments were conducted by providing two separate signals at two different frequencies to the speaker and the electrode. In the framework of this operational scenario, the frequency of a voltage signal supplied to the electrode is swept in the vicinity of twice the beam resonant frequency, such that $\omega_{p}\approx 2\omega_{e}$, which allows us to hinder the influence of the “parasitic” electrostatic excitation. Figure 8 presents the results of the cantilever response in the case of the single- and double-frequency purely electrostatic $V_{spkr}=0$ excitation. The beam was first operated by the signal with $V_{dc}=0$ and $V_{ac}\approx 40$ $V_{pk-pk}$, and the excitation frequency $\omega_{p}$ was swept between ≈40 kHz and ≈50 kHz. In another scenario, the voltages were $V_{dc}\approx 20$ V, $V_{ac}\approx$ 40 $V_{pk-pk}$ and the frequency range was between ≈80 KHz and ≈100 KHz. In both cases, the response is solely due to the parasitic electrostatic excitation. As suggested by Figure 8a depicting the time histories, the amplitude is more than 80 times higher in the case of the single-frequency excitation. The presence of two frequencies is observed in the time history corresponding to the double-frequency excitation, indicating that the response is not resonant. Since the voltage modulation was below the parametric resonance threshold, the Lorentzian frequency curves were obtained in both cases, Figure 8b. Note that as these frequency curves were obtained using a real-time spectrum analyzer, the frequency axis in Figure 8b corresponds to the actual frequencies of the beam’s vibratory response rather than the frequency of the driving signal.

**Figure 9:**
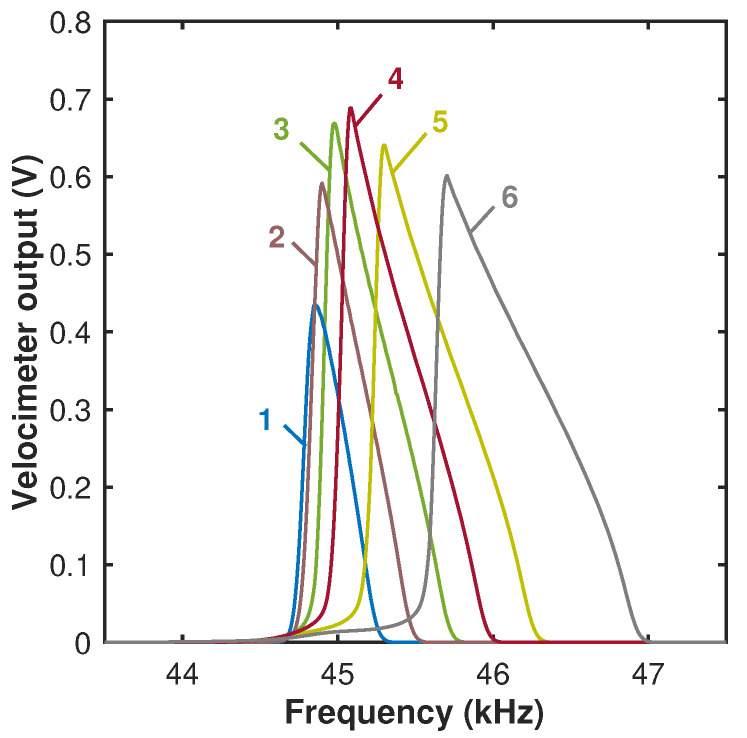
Experimental frequenting curves illustrating parametric resonance for the case of pure electrostatic excitation at $\omega_{p}\approx 2\omega_{e}$. Frequency response above the PR threshold at different $V_{ac}$ values, $V_{dc}\approx 20$ V. Curve 1 corresponds to $V_{ac}\approx 40$ V; curve 2 corresponds to $V_{ac}\approx 50$ V; curve 3 corresponds to $V_{ac}\approx 60$ V; curve 4 corresponds to $V_{ac}\approx 70$ V; curve 5 corresponds to $V_{ac}\approx 80$ V; curve 6 corresponds to $V_{ac}\approx 100$ V. The frequency axis corresponds to the frequencies of the beam’s vibratory response.

To illustrate the excitation of the parametric resonance and to estimate the ac voltage corresponding to the parametric resonance threshold, a harmonic voltage signal at the frequency equal to twice the effective resonant frequency, $\omega_{p}\approx 2\omega_{e}$, was applied to the electrode. The speaker voltage was zero in this experiment. In this case, the beam does not undergo any acoustic or “parasitic” direct excitation at the fundamental mode effective resonant frequency $\omega_{e}$ [16]. Results corresponding to $V_{dc}\approx 20$ V and various $V_{ac}$ values above the parametric threshold are shown in Figure 9. Also here, the frequency axis corresponds to the actual frequencies of the beam’s vibratory response.

**Figure 10:**
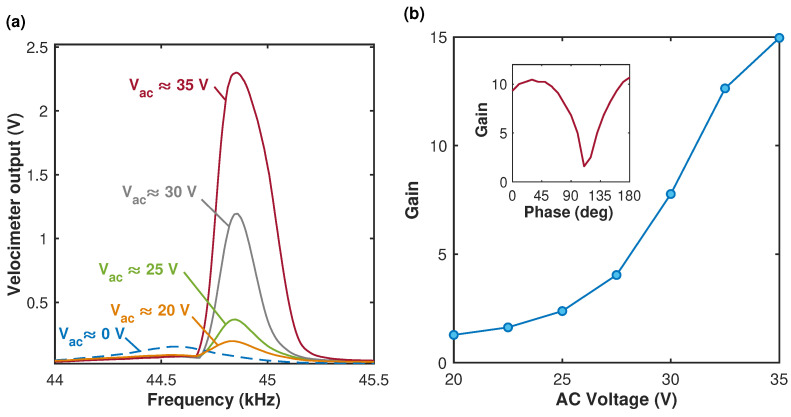
Parametric amplification below the PR threshold. (**a**) $V_{spkr}\approx 5$$V_{pk-pk},V_{dc}\approx 20$ V. (**b**) Gain. $V_{spkr}\approx 5V_{pk-pk},V_{dc}\approx 20$ V, and ϕ≈0. The frequency of the electrostatic signal is $\omega_{p}=2\omega_{e}$, the frequency axis corresponds to the frequencies of the beam’s response. Inset illustrates a dependence of the gain on the phase between two signals at $V_{ac}\approx 30$ V.

Finally, in the framework of the parametric amplification experiment, the harmonic voltage at the beam’s effective resonant frequency ($\omega \approx \omega_{e}$) was applied to the speaker while the electrode was harmonically driven at $\omega_{p}\approx 2\omega_{e}$ and $V_{ac}$ below the PR threshold value. The phase between the two signals in the wave generator was set to zero. Due to different conversions of the original electric signals into the mechanical forces associated with the acoustic pressure and the electrostatic field, the actual phase between the two (electrostatic and acoustic) forces differs from the phase between the voltage signals supplied to the speaker and the electrode. Since the measurement of the actual phase between the direct drive and the parametric amplification is challenging, we consider the phase between the corresponding (supplied to the electrode and the speaker) voltage signals as a control parameter. The measured frequency responses acquired at various $V_{ac}$ values are shown in Figure 10a, and the corresponding parametric gain is depicted in Figure 10b. The experimental results are in qualitative agreement with the SDOF model predictions (Figure 2). We also investigated how the phase difference between the signals supplied to the speaker and to the electrode affects the parametric amplifier gain. The result is shown in the inset in Figure 10b. The asymmetry in the gain–phase curve, which is attributed to the presence of nonlinearity in the system, is consistent with the model predictions shown in Figure 3. Note that apart from the parametric amplification, the change in the response amplitude may occur due to the influence of nonlinearities as well as the direct parasitic electrostatic driving. Our calculations show that for the beam’s amplitude well below the cantilever’s thickness the contribution of the nonlinearities compared to the high parametric gain (up to 15 at $V_{ac}\approx 35$ V) is small. As suggested by Figure 10a, the influence of nonlinearities starts to have certain influence at $V_{ac}$ above 30 V. While the direct electrostatic driving due to the parasitic fringing fields could be present (as suggested by Figure 7), its contribution is negligible since the excitation is at twice the effective resonant frequency and the direct electrostatic excitation is nonresonant. Indeed, the results shown in Figure 7 and Figure 8 collectively suggest that the magnitudes of the responses due to the parasitic electrostatic excitation in the parametric amplification experiment, Figure 10, are expected to be orders of magnitude lower than that of the acoustic signal. The dependence of the parametric gain on the pumping voltage and the phase observed in our experiments is typical for electrostatically driven parametrically amplified devices [1,4].

## 4. Conclusions

In this paper, we demonstrate theoretically and experimentally that electrostatic excitation by fringing fields can be efficiently used for the upward frequency tuning and parametric amplification of acoustically excited microstructures. This approach can be beneficial in acoustic sensors. It contributes also to a better understanding of the influence of the environmental acoustic fields on the dynamic behavior of microsensors. Note that in the present work, the experiments were conducted at frequencies in the vicinity of 45 kHz, mainly for the sake of convenience and compatibility with the spectral contents of the characterized MEMS devices. While the range of low-frequency ultrasonics can be relevant for acoustic sensors or for biological signals acquisition and analysis (such as bat echolocation calls [40]), the same approach can be used for the performance enhancement of the devices operated in an audible range, such as hearing aids. In addition, the noncontact acoustic force has been demonstrated as a useful and convenient experimental technique for dynamic characterization of unpackaged MEMS devices. We believe that the same approach can be used for efficient wafer-level mechanical testing and early-stage functionality screening of microstructures.

## Figures and Tables

**Table 1:** Nominal (as designed) and measured (mean ± standard deviation) beam parameters (in µm) used in the experiments. The estimated measurement error was based on the pixel-to-µm conversion factor of the scanning electron micrographs.

Parameter	Nominal	Measured
Length of the beam *L*	300	≈296.13 ±0.64
Width of the beam *b*	16	≈15.13 ±0.64
Thickness of the beam *d*	3	-
Distance to electrode $g_{0}$	5	≈5.61 ±0.64
Length of the electrode $L_{e}$	100	≈97.27 ±0.64

**Table 2:** Nondimensional quantities. Derivations of the values can be found in [35].

Notations	Parameter
$q=\hat{q}/d$	Deflection
$t=\hat{t}\sqrt{EI/(\rho AL^{4})}$	Time
$\omega =\hat{\omega }\sqrt{\rho AL^{4}/\left(EI\right)}$	Frequency
$\beta =asL^{4}V_{0}^{2}/\left(EIdm\right)$	Voltage parameter

**Table 3:** Parameters used in calculations. The electrostatic force fitting parameters are adopted from [37].

Parameter	Value
Density ρ (kg/$m^{3}$)	2300
Young’s modulus *E* (GPa)	169
Modal electrostatic force parameter *s*	0.205
Modal mass *m*	0.25
Modal coeff. of the acoustic force η	0.0037
Electrostatic force fitting parameter *a*	$2.15\times 10^{-6}$
Electrostatic force fitting parameter σ	1.05
Electrostatic force fitting parameter *p*	1.4

## Data Availability

Data are contained within the article.
